# Evaluating the Well-Being Benefits and Social Value of Volunteer Gardening: Health Economics Meets Behavioral Science

**DOI:** 10.3390/bs14121233

**Published:** 2024-12-21

**Authors:** Holly Whiteley, John Parkinson, Ned Hartfiel, Abraham Makanjuola, Huw Lloyd-Williams, Catherine Lawrence, Rhiannon Tudor Edwards

**Affiliations:** 1 Centre for Health Economics and Medicines Evaluation, Bangor University, Gwynedd LL57 2PZ, UK; ned.hartfiel@bangor.ac.uk (N.H.); c.l.lawrence@bangor.ac.uk (C.L.); r.t.edwards@bangor.ac.uk (R.T.E.); 2 Wales Centre for Behaviour Change, Department of Psychology, Bangor University, Gwynedd LL57 2AS, UK; j.parkinson@bangor.ac.uk; 3 Faculty of Life Sciences and Education, University of South Wales, Pontypridd CF37 4BD, UK; abraham.makanjuola@southwales.ac.uk; 4 Wavehill Social and Economic Research, Aberaeron, Ceredigion SA46 0DB, UK

**Keywords:** health economics, behavioral science, social value, social cost–benefit, illness prevention, well-being, mental health, psychological well-being, gardening, volunteering

## Abstract

Multidisciplinary collaboration is key to strengthening the evidence base for multifaceted illness prevention interventions. We bring together health economics and behavioral science to explore the well-being benefits and social cost–benefit of volunteer gardening at an accredited botanic garden, Wales, UK. A health economics-informed social return on investment (SROI) evaluation was combined with the assessment of volunteers’ basic psychological needs (autonomy, competence, and relatedness), connection to nature, and their interrelatedness in this innovative nature-based intervention study. Pre- and post-volunteering outcome data were collected using the Short Warwick-Edinburgh Mental Well-being Scale (SWEMWBS), the ICEpop CAPability measure for Adults (ICECAP-A), the 12-item diary version of the Basic Psychological Need Satisfaction and Frustration Scale (BPNSNF), the Nature Connection Index (NCI), and a bespoke Client Service Receipt Inventory (CSRI). Results indicate that volunteer gardening can provide well-being benefits to participants and cost savings to the NHS. The well-being benefits observed were estimated to generate social value in the range of GBP 4.02 to GBP 5.43 for every GBP 1 invested. This study contributes to the evidence base that simple nature-based interventions such as volunteer gardening could offer low-cost supportive environments that deliver significant well-being benefits and associated social value to local communities, including a reduced burden on overstretched local healthcare services.

## 1. Introduction

Prevention interventions to address complex societal challenges, such as the current mental health crisis [1,2,3], are often multifaceted and can yield a diversity of benefits [4]. Nature-based interventions (NBIs) that provide access to non-clinical activities in natural settings [5], for example, tend to involve multiple components, require co-production with participants, and can deliver a wide range of health, well-being, and social outcomes [6,7].

NBIs such as woodland therapy, therapeutic horticulture, and ecotherapy can improve feelings of life satisfaction and happiness and could result in health system cost savings of GBP 800 to GBP 1500 per person over one year [8]. NBIs involving regular gardening have been found to reduce perceived stress, anxiety, and depression and promote mental well-being [9,10,11,12,13], with those who garden daily found to have a 6.6% higher well-being and 4.2% lower stress levels compared to non-gardeners [14]. Community gardens which provide access to green space and bring members of the local community together have been found to have a wide range of positive well-being and social impacts, such as improving physical and mental health, neighbourhood safety, social cohesion, and a sense of belonging [15,16,17]. It is estimated that GBP 2.1 billion per year could be saved in healthcare costs if residents in England had good access to green space [17,18]. 

Volunteering has also been found to positively impact well-being, particularly for older adults, through the social interaction and sense of purpose it provides [19,20,21,22,23]. The National Council for Voluntary Organisations, for example, reported that 77% of volunteers reported improved mental well-being and 53% experienced improved physical health, and that volunteering helped people form new friendships and combat feelings of isolation [24]. In a longitudinal study of adults aged 50 and over in the United States, Kim et al. (2020) found that those who volunteered for more than 100 h per year had a lower risk of mortality and physical limitations and higher levels of physical activity and better psychosocial outcomes than those who did not volunteer [25]. 

In terms of the potential mechanisms driving the well-being benefits of NBIs and volunteering, immersion in purposeful practical work has been seen to help people experience a sense of flow and meaning and disconnect from concerns and worries [21,26,27]. Learning new skills can help build self-confidence and self-worth [26], and volunteering can enable the satisfaction of core psychological needs such as competence, autonomy, and relatedness [28], particularly with respect to autonomous forms of regulation and motivation [29]. There is a growing body of work exploring the health and well-being benefits of contact and proactive interaction with nature [6,7,26,30,31]. Some studies have indicated that simply walking in nature can facilitate attentional rest and help reduce negative rumination, supporting mental health [32,33].

Supporting the effective and cost-effective real-world implementation of complex illness-preventation interventions such as NBIs requires a broad multidisciplinary approach that considers all impacts and the mechanisms through which they are realised, and generates appropriate cost–benefit evidence [4]. Here, we apply health economics, a mixed-method SROI evaluation, and an exploration of psychological need fulfilment in a collaborative and innovative study to explore the well-being benefits and associated social value of volunteer gardening at Treborth Botanic Garden (TBG), an accredited botanic garden in Wales, UK.

## 2. Materials and Methods

### 2.1. Study Setting

Under the ownership of Bangor University since 1960, TBG comprises 15 hectares of native woodland, 2 hectares of rich, species-diverse grassland, and 1 hectare of managed orchard. TBG is also home to six glasshouses, each with regulated temperatures to accommodate special collections of exotic flora, such as orchids and cacti. The garden is recognised as an accredited botanic garden by Botanic Garden Conservation International (BGCI), making it one of seven accredited botanic gardens in the UK and one of only three accredited botanic gardens in Wales.

TBG is committed to fostering community engagement, running a local volunteer programme and hosting a diverse array of events and workshops for the public. The gardens are free and open to the public throughout the year and the glasshouses can be accessed during specific times when staff or volunteers are available. TBG attracts approximately 35,000 visitors each year. 

The maintenance and development of TBG is managed by a team of three members of Bangor University staff supported by local volunteers via the TBG staff-run volunteer programme. Volunteers include members of Friends of Treborth Botanic Garden (FoTBG), a charitable organisation, and the Students for Treborth Action Group (STAG). Volunteers assist with a variety of tasks, including the maintenance of the gardens, woodland, and glasshouses, non-horticultural maintenance, event support (e.g., plant sales), and representing TBG at external events.

### 2.2. Study Design

This study was based on a natural experiment (NE) study design [34] and health economic-informed SROI evaluation methodology. Due to the opportunistic and real-world scenario nature of the study, there was no control group available and blinding amongst researchers and participants was difficult. The study received ethics approval from the Bangor University Medical and Health Sciences Academic Ethics Committee (reference number: 2021-17011; 15 November 2021). 

### 2.3. Participants

All volunteers at TBG (approx. 96 volunteers in 2021/2022) were invited to take part in the study in November/December 2021, including existing volunteers who had started volunteering before November 2021 and new Bangor University staff and student volunteers who started volunteering in the first term of the academic year 2021/2022. All TBG volunteers received a participant information sheet (PIS) that introduced the study and provided key information about participation, along with an informed consent form. The TBG Curator and Student Volunteer Coordinator informed volunteers of the study and made hardcopy versions of the PIS and consent form available to volunteers attending the garden in November/December 2021. Electronic versions of the PIS and consent form were also circulated to all volunteers at the start of the study via the volunteer programme mailing list, managed by the TBG Curator. 

Volunteers were eligible for participating in the study if they met the following criteria:

- Were aged over 18 years;
- Had the capacity to give informed consent to take part in the evaluation;
- Were able to speak, read, and write in English or Welsh.

### 2.4. Data Collection

Data collection took place between November 2021 and April 2022. Pre-volunteering (before starting to volunteer at TBG) and post-volunteering ( ‘follow-up’ (April 2022 outcome data were collected from study participants using questionnaires containing the following validated measures: the Short Warwick-Edinburgh Mental Well-being Scale (SWEMWBS), the ICEpop CAPability measure for Adults (ICECAP-A), the 12-item diary version of the Basic Psychological Need Satisfaction and Frustration Scale (BPNSNF), the Nature Connection Index (NCI), and a bespoke Client Service Receipt Inventory (CSRI). Further information about each outcome measure is provided in Section 2.6.3 and 2.7 below. New staff and student volunteers completed outcome questionnaires at the study baseline (November 2021) and the six-month follow-up (April 2022). Existing volunteers completed a ‘one-time’ questionnaire in April 2022 that included retrospective baseline and current day follow-up versions of each validated measure. On average, the questionnaires took participants approximately ten minutes to complete. 

### 2.5. Social Return on Investment (SROI) Methodology

The National Institute for Health and Care Excellence (NICE) and HM Treasury’s Green Book recommend the use of CBA and social CBA for evaluating the cost-effectiveness of complex public health and well-being interventions. The SROI is a pragmatic form of social cost–benefit analysis (CBA) that can explore the economic, environmental, and social costs and benefits of an organisation’s activities from the perspective of the people and organisations that benefit from them [35,36].

The SROI considers the costs and outcomes of an activity or intervention that are relevant and significant to stakeholders and assigns a market or financial proxy value to these. Well-being-related outcomes can be monetised using a well-being valuation, which provides a consistent and robust method for estimating the monetary value of outcomes that do not have market values. Recommended in the HM Treasury Green Book, a well-being valuation uses thousands of large UK national surveys to isolate specific variables and to determine the effect of those variables on a person’s well-being [33]. A well-being valuation establishes the equivalent amount of income needed to increase a person’s well-being by the same amount. 

A well-being valuation can be applied using two social value calculators developed by the Housing Associations’ Charitable Trust (HACT), a UK charity of the social housing sector www.hact.org.uk (accessed on 5 October 2021). These include the HACT Social Value Calculator (SVC) derived from the HACT Social Value Bank (SVB), and the HACT Mental Health Social Value Calculator (MHSVC) that estimates the health-related social value from reported changes in Short Warwick-Edinburgh Mental Well-being Scale (SWEMWBS) scores [37]. Both calculators value similar elements of health and well-being and are therefore used separately to avoid double-counting. In this study, we applied a well-being valuation using both the SVC and MHSVC to generate a range of SROI ratios, serving as a form of embedded sensitivity analysis and helping to verify the results. These SROI ratios compare the costs of supporting volunteer gardening at TBG with the monetised benefits experienced by volunteers and other key stakeholders using the below formula:

$$SROI\;ratio=\frac{Social\;value\;of\;stakeholder\;outcomes}{Cost\;of\;faciliting\;volunteering\;at\;TBG}$$

### 2.6. Stages of SROI Analysis

The main stages of SROI analysis include identifying stakeholders, developing a logic model to identify key stakeholder outcomes, evidencing outcomes, valuing outcomes, calculating costs, and estimating the SROI ratio.

#### 2.6.1. Identifying Stakeholders

Stakeholder identification and involvement is essential to the effective design and conduct of SROI evaluations [35]. The main stakeholders identified in this SROI evaluation are listed below (Table 1).

There were approximately 96 volunteers registered with the TBG volunteer programme in 2021/2022. These volunteers were divided into three groups:

1. Existing volunteers: This group included approximately 70 locally employed and retired people (approximately 73% of the total TBG volunteer population).
2. Bangor University student volunteers: This group included approximately 20 students (approximately 21% of the total TBG volunteer population) who attended a monthly work party.
3. Bangor University staff volunteers: This group included approximately 6 members of university staff (approximately 6% of the total TBG volunteer population) that spent half a day per month at TBG learning gardening skills and participating in seasonal garden projects.

#### 2.6.2. Developing a Logic Model

**Figure 1:**
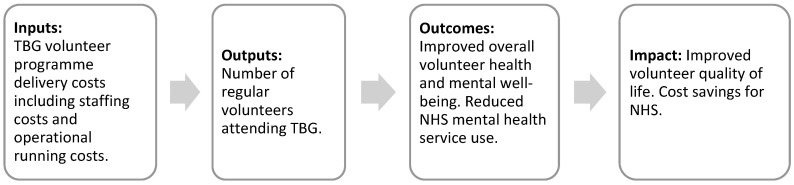
Logic model.

Logic models illustrate the underlying linkages between the inputs, outputs, and expected outcomes of a programme. A logic model was developed to explore how voluntary gardening inputs (i.e., the costs of facilitating and enabling volunteering) were converted into outputs (i.e., the number of volunteering hours) and subsequently into stakeholder outcomes to estimate the social value generated (Figure 1).

#### 2.6.3. Evidencing Outcomes for Well-Being Valuation

Two validated outcome measures were included in questionnaires to assess the health and well-being of TBG volunteers (Table 2). The ICEpop CAPability measure for Adults (ICECAP-A) is a reliable capability-based 5-item health and well-being measure for the general adult (18+) population [38,39]. It encompasses a broad definition of health and well-being by including five attributes of well-being that were found to be important to adults in the UK. These attributes include (i) feeling settled and secure, (ii) love, friendship, and support, (iii) being independent, (iv) achievement and progress, and (v) enjoyment and pleasure. A total tariff value reflecting an overall health-related quality-of-life state is calculated for all five attributes. These total tariff values can range from −0.001 to 1, with 1 reflecting full capabilities or health. The ICECAP-A measure was used to assess overall volunteer health pre- and post-volunteering.

The 7-item Short Warwick-Edinburgh Mental Well-being Scale (SWEMWBS) is a shortened version of the Warwick-Edinburgh Mental Well-being Scale (WEMWBS) that is validated and reliable for a range of UK populations and settings [40,41]. The SWEMWBS contains seven statements with five response groupings linked with characteristics of positive mental health and has a score range of 7 to 35, with 7 reflecting very poor mental health and 35 reflecting excellent mental health [42]. Here, we used the SWEMWBS to measure volunteer mental well-being pre- and post-volunteering.

A bespoke Client Service Receipt Inventory (CSRI) asking participants about the number of appointments with NHS mental health services was also included in questionnaires to assess potential changes to volunteer NHS mental health service use pre- and post-volunteering.

#### 2.6.4. Monetising Outcomes for Well-Being Valuation

In this SROI evaluation, the social value was estimated using the SVC, MHSVC, and national unit costs of NHS mental health service use (Table 2). For the SVC-based well-being valuation, financial proxies from the HACT Social Value Bank v4 [43] were used to assign monetary values to the outcomes of ‘regular volunteering’ and ‘good overall health’. The SVB proxy value of GBP 20,791 for ‘good overall health’ was applied to volunteers who reported a ≥10% improvement in ICECAP-A scores between the baseline and follow-up, following a similar approach to Hartfield et al. (2023) [44]. The SVB proxy value of GBP 5344 was applied to participants who volunteered regularly (i.e., at least once per month for two months). To complement the above SVC approach, the MHSVC well-being valuation derived financial proxies from changes in volunteer SWEMWBS scores pre- and post-volunteering.

Finally, to explore the social value generated for NHS Wales by volunteer gardening at TBG, the national unit cost of NHS mental health services in 2022 were used to estimate the social value generated by the change in the number of volunteer visits to psychotherapists, counselling, psychologists, and mental health nurses pre- and post-volunteering (Table 2) [45,46].

**Table 2:** Well-being valuation outcomes and outcome measures.

Stakeholder	Outcomes	Outcome Measures	Well-Being Valuation Method	Financial Proxy	Financial Proxy Source
Volunteers	Regular volunteering	Regular volunteering involves volunteering at least once a month for a minimum of two months	SVC	GBP 5344 per person per year	Social Value Bank v4 [43]
	Good overall health	ICECAP-A	SVC	GBP 20,791 per person per year	Social Value Bank v4 [43]
	Mental well-being	SWEMWBS	MHSVC	Varies depending on total SWEMWBS score	Mental Health Social Value Calculator v1 [37]
NHS Wales	Visits to NHS mental health services	Bespoke Client Service Receipt Inventory (CSRI) asking participants about the number of appointments with NHS mental health services	National unit cost	Psychotherapist: GBP 216 per visit	Personal Social Services Research Unit (PSSRU) [45]
				Counselling: GBP 75 per visit	NHS England [46]
				Psychologist: GBP 125 per visit	NHS England [46]
				Mental health nurse: GBP 21 per visit	NHS England [46]

#### 2.6.5. Valuing Outcomes Using the SVC

When using the SVC for well-being valuation, the SROI methodology requires that the deadweight, attribution, and displacement are considered to prevent overclaiming the amount of social value generated by a programme or activity. The deadweight reflects the possibility that a proportion of the outcomes may have happened anyway without the programme; attribution acknowledges that a proportion of the outcomes may be attributable to factors other than the programme; and displacement considers whether participants had to give up any other activities from which they might have benefitted. The below follow-up questions were included in questionnaires to assess the deadweight, attribution, and displacement:

Deadweight: ‘How much of this change do you think would have happened anyway, if you hadn’t participated in the TBG volunteering?’ 

Attribution: ‘How much of the change do you think is due to the Treborth programme?’

Displacement: ‘By participating in TBG volunteering, how much have you had to give up other activities that benefitted you?’

#### 2.6.6. Valuing Outcomes Using the MHSVC

After a total SWEBWBS score was recorded for each participant pre- and post-volunteering, a monetary value was assigned to each total score using the MHSVC financial proxies [37]. The pre-volunteering monetary value was then subtracted from the post-volunteering monetary value for each participant. A standard deadweight percentage of 27% for health interventions was then subtracted to calculate the total social value for each participant [37].

#### 2.6.7. Calculating Costs

TBG volunteer programme delivery costs included staffing costs and running costs. Staff costs were estimated based on Bangor University salary scales. TBG volunteer programme running costs were estimated by apportioning a percentage of the total TBG operational running costs towards the volunteer programme. These cost estimates were provided by TBG staff. 

#### 2.6.8. Estimating the SROI Ratio

SROI ratios were calculated by comparing the total costs with the monetised outcomes calculated using the SVC, MHSVC, and NHS national unit costs.

### 2.7. Additional Volunteer Outcomes

Alongside outcomes for inclusion in the well-being valuation for SROI estimation, volunteers’ basic psychological needs and connection to nature were also explored.

#### 2.7.1. Basic Psychological Needs and Nature Connection

Volunteers’ basic psychological needs were assessed using the validated Basic Psychological Need Satisfaction and Frustration Scale (BPNSFS) [47,48]. To reduce the questionnaire burden and fatigue of participants, the 12-item diary English version of the BPNSFS was used. The BPNSFS is based on Basic Psychological Need Theory and assesses the extent to which the psychological needs for autonomy, competence, and relatedness are met (satisfaction) or not met (frustration). Whilst a lack of need satisfaction does not imply need frustration, need frustration does imply low need satisfaction [49]. Changes in volunteer-reported psychological need satisfaction and psychological need frustration were assessed separately. 

Volunteers’ connection to nature was measured using the validated Nature Connection Index (NCI) [50]. Total scores on the NCI range from 0 to 100, with 0 reflecting no connection to nature and 100 reflecting a strong connection to nature. Changes in volunteers’ basic psychological need satisfaction and frustration and connection to nature between the baseline and follow-up are reported alongside the SROI results.

#### 2.7.2. Data Analysis

To accompany the mixed-method SROI evaluation in this study, simple statistical analyses on volunteer-reported outcomes were undertaken to explore the differences pre- and post-volunteering, as well as the interrelatedness between them. A nonparametric sign test for two related samples was used to compare participant baseline and follow-up (pre- and post-volunteering) ICECAP-A, SWEMWBS, BPNSFS, and NCI scores. The nonparametric Spearman rank correlation coefficient was also applied to explore the correlation between these outcomes. All statistical analyses were conducted in IBM SPSS Statistics Version 29.0.1.0. 

## 3. Results

Thirty-five out of a total of ninety-six TBG volunteers completed the baseline and follow-up (pre- and post-volunteering) questions (a 37% response rate). Baseline demographic characteristics for these participants are presented in Table 3. The proportional representation of existing volunteers, Bangor University student volunteers, and Bangor University staff volunteers was 74%, 20%, and 6%, respectively. The study sample is therefore considered to be proportionally representative of the total TBG volunteer population (see Section 2.6.1).

### 3.1. Reported Outcomes

Changes in volunteer well-being outcomes are reported in Table 4. Overall, volunteers reported an improvement in overall health (ICECAP-A mean % difference of +9 ± 14 SD) and mental well-being (SWEMWBS mean % difference of +9 ± 16 SD), and reduced basic psychological need frustration (BPNSFS mean % difference of −11 ± 17 SD). Small increases in mean basic psychological need satisfaction and nature connection were also reported.

### 3.2. Statistical Analysis of Outcomes

The related sample sign test results indicate a significant difference in volunteer-reported ICECAP-A and SWEMSBW scores pre- and post-volunteering (*p*-value < 0.001), suggesting a significant improvement in volunteer overall health and mental well-being (Table 5). There was also a significant difference (*p*-value of 0.012) in the volunteer-reported basic psychological need frustration, indicating an improvement in psychological well-being due to a perceived reduction in the violation or loss of autonomy, competence, and relatedness. Positive changes in the sense of autonomy and competence, in particular, seemed to drive this difference pre- and post-volunteering.

The exploration of the interrelatedness between the above volunteer outcomes at the follow-up using Spearman’s rank correlation found a significant positive correlation between SWEMWBS and basic psychological need satisfaction scores (r = 0.634; *p*-value ≤ 0.001) and a significant negative correlation between SWEMWBS and basic psychological need frustration scores (r = −0.514; *p*-value = 0.009). A negative correlation was also found between basic psychological need satisfaction and frustration scores at the follow-up (r = −0.623; *p*-value ≤ 0.001).

### 3.3. Monetising Well-Being Outcomes

The total social value per volunteer estimated using the SVC was GBP 16,750 before considering the deadweight, attribution, and displacement (Table 6). 

With respect to the estimation of the deadweight, TBG volunteers indicated that 10% of the change they experienced would have happened anyway. TBG volunteers also indicated that 57% of the change they had experienced occurred due to the programme (attribution) and that they had to forego 43% of other supportive, beneficial activities (displacement). When the above estimates for the deadweight, attribution, and displacement were considered, the total social value per volunteer per year was GBP 3695 (Table 7).

In comparison, the application of the MHSVC to volunteers’ SWEMWBS scores gave us an estimated total social value of GBP 2684 per volunteer per year (Table A1).

### 3.4. NHS Mental Health Resource Use

Overall, TBG volunteers reported using less psychotherapist, counselling, and mental health nurse services when they were volunteering compared to the period before they started volunteering (Table 8). In contrast, the average number of visits to a psychologist increased. This change in the average psychologist visits was driven by the service use of a very small number of volunteers and may not be representative of the whole study sample or volunteer population.

### 3.5. Valuing Inputs

TBG staff were consulted to determine the total operational costs of running TBG, including staffing and annual running expenses, and the proportion of these costs that could be attributed to facilitating the TBG volunteer programme.

#### 3.5.1. Staffing Costs

TBG employed three full-time-equivalent (FTE) Bangor University staff members at an average annual salary of GBP 27,396 or GBP 14.53 per hour. Staffing costs for volunteering included the number of hours spent each year on recruiting, planning, and managing volunteers, which TBG staff estimated to be 8 hours per day, 160 h per month, and 1920 h per year on average. Three FTE staff members worked an average of 7.25 h per day, which totalled to 21.75 h. The eight hours spent on volunteering tasks each day represented 37% of the total staff time.

#### 3.5.2. Running Costs

The running costs consisted of ongoing expenses like the building lease, maintenance, utilities, equipment, materials, catering, and insurance. Since 37% of staff costs were attributed to volunteers, we apportioned 37% of operational expenses to facilitating the TBG volunteer programme. The cost per volunteer was estimated based on a total of 96 volunteers registered on the TBG volunteer programme in 2021/2022 (Table 9).

### 3.6. Calculating the SROI Ratio

SROI ratios were calculated using the SVC and the MHSVC, both incorporating the national unit costs of NHS mental health service use. When the total social value per volunteer was compared with the total cost per volunteer, the SROI ratios ranged from GBP 4.02 to GBP 5.43 for every GBP 1 invested (Table 10).

## 4. Discussion

Although the body of evidence regarding the health and well-being benefits of regular gardening and volunteering is expanding, little is known of their social cost–benefit and the mechanisms behind their positive impact. This information is essential for supporting the real-world implementation of these multifaceted illness prevention interventions. Quantitative data from this study indicate that many TBG volunteers experienced improvements in overall health (23 out of 33 participants) and mental well-being (27 out of 35 participants), and reported reduced levels of basic psychological need frustration (21 out of 32 participants). This is supported by the significant statistical differences pre- and post-volunteering indicated by our statistical analyses. Some volunteers also reported small positive changes in basic psychological need satisfaction (16 out of 32 participants) and reported a greater connection to nature (10 out of 34 participants), but no significant difference was observed between pre- and post-volunteering for these outcomes. Whilst the small, non-significant change in the nature connection may seem surprising in a nature-based intervention such as volunteer gardening, it may be that this voluntary activity attracted individuals who already had a good connection to nature. This is reflected in the mean NCI score of 75 out of 100 at pre-volunteering baseline.

The significant correlations between SWEMWBS and basic psychological need satisfaction and frustration scores indicate the close relationship between mental well-being and the fundamental human need for autonomy, competence, and relatedness [51,52]. The negative correlation between need satisfaction and frustration was expected given the asymmetrical relationship between the two [49].

The SROI evaluation based on volunteer-reported changes in ICECAP-A and SWEMWBS measures used to assess overall health and mental well-being, respectively, and the reported changes in NHS mental health services use, indicates that the TBG volunteer programme generated positive social value ratios in the range of GBP 4.02 to GBP 5.43 for every GBP 1 invested in 2021/2022. This is greater than the social value estimated by a recent SROI evaluation of a community garden in London, which revealed that for every GBP 1 invested, GBP 3 of social value was generated, based on the increased confidence, social isolation, and emotional well-being of garden users and reduced hospital admissions [53].

The increased capability and psychological well-being reflected in the improved ICECAP-A, SWEMWBS, and basic psychological need scores (especially in terms of autonomy and competence) may reflect that immersion in purposeful practical work enabled TBG volunteers to learn, develop, and maintain new and existing skills [21,26,27], as well as exercise their autonomous motivation in well-being-promoting activities [28,29]. 

### 4.1. Strengths of This Study

This study used well-known, validated, and reliable measures for assessing changes in overall health and mental well-being (ICECAP-A and SWEWBS) and validated measures for exploring basic psychological needs (BPNSFS) and nature connections (NCI). We also utilised two well-established well-being valuation approaches using the SVC derived from the HACT Social Value Bank (SVB) [43] and the MHSVC derived from the SWEMWBS [37]. These two well-being valuation approaches were used separately alongside each other to avoid double-counting and give robustness to the SROI evaluation results. 

### 4.2. Limitations of This Study

This SROI evaluation focused on two stakeholder groups, TBG volunteers and NHS Wales. Volunteer gardening at TBG may have impacts for other stakeholders, for example, Bangor University and the environment. The collection of this wider data, however, was outside the scope of this study and may have resulted in an underestimation of the total social value generated by the TBG volunteer programme. In contrast, the nature of this small-scale natural experiment(NE)-based study, the low volunteer response rate and small sample size (complete cases: N = 35 out of 96 TGB volunteers), and lack of a control group means that the ability to generalise the participant-reported outcomes and estimated social value to the wider population is limited. The true return on investment at a scale across the total population is uncertain and may be lower than the social cost–benefit presented here.

There may be differences in the outcomes and social value generated between the three groups of TBG volunteers included in this analysis (existing volunteers, student volunteers, and staff volunteers). There may also be differences in the experience of outcomes between volunteers with different experiential time frames, i.e., the total amount of time spent volunteering and the frequency and duration of volunteer sessions. Robust subgroup analysis to explore individual differences was not possible due to the small sample size, especially with the limited amount of complete case data for student and staff volunteers.

The small sample size of this study may have resulted from the lack of an incentive offered to volunteers to encourage participation and/or the time of year at which the study was undertaken (November to April, when volunteers may be less active at TBG during winter months). The sampling strategy may also have influenced the nature of volunteers who chose to participate in the study, which could bias the results. 

The lack of a comparator intervention or control group is a common limitation shared by other nature-based interventions [54], making it difficult to ascertain causality. We acknowledge that the benefits of volunteering, gardening, and simply spending time in nature are likely to overlap and that there are wide range of potential mechanisms by which contact with nature can influence health [31]. The investigation of causality and mechanisms of change would require a more controlled and rigorous study design and analysis than this small-scale NE-based study allowed for.

The small sample size, 6-month time horizon of the study, and lack of a control group made it difficult to consider potential confounding variables, such as different experiential time frames and the time of year/season. The application of the deadweight, attribution, and displacement in the SROI methodology, however, helped to mitigate against overclaiming the positive social impact directly attributed to the TBG volunteer programme.

### 4.3. Future Research

Robust quantitative evidence of the well-being benefits of regular volunteer gardening, particularly regarding mental health service use, cost savings for the NHS, and the mechanisms of change behind these outcomes, would support the integration of this simple, low-cost intervention into policy and practice, i.e., through green social prescribing. 

Future evaluations to explore well-being benefits and the social cost–benefit of NBIs such as volunteer gardening would benefit from better controlling for confounding factors and exploring causality in greater depth. This could be achieved by studying change over longer time horizons, i.e., a minimum of 12 months to account for seasonal variation, improving sample sizes, and using more controlled, rigorous study designs and statistical analysis that allow for the investigation of individual differences, including how different people or groups might fulfil different psychological needs (i.e., autonomy, competence, and relatedness) through participation in the same activity. The above would ultimately improve our understanding of the extent to which observed health and well-being benefits are a result of gardening and/or volunteering activity and provide greater insight into the mechanisms of change behind these benefits.

We believe that an SROI underpinned by health economics, behavioral science, and a robust study design provides a useful approach for evaluating multifaceted illness prevention interventions that have the potential to generate a range of health and well-being-related outcomes across different groups of stakeholders.

## 5. Conclusions 

The findings of this study indicate that NBIs such as volunteer gardening can generate a positive social value through the improvement of local community mental health and reduce pressure on local healthcare systems. This evidence could help encourage further research into the well-being benefits of volunteering and gardening and the ongoing sustainable funding and support for volunteer gardening initiatives going forwards.

Multidisciplinary collaboration is key to strengthening the evidence base for effective and cost-effective illness prevention strategies. Viewing a problem from different perspectives, and through different world view lenses, helps to develop a shared understanding. Similarly, such an approach provides convergent evidence that is more robust and meaningful when identifying solutions and implementation actions [55]. Expanding our multidisciplinary approach to assess the wider benefits to other stakeholders and the potential environmental outcomes of volunteer gardening would further improve understanding of its diverse impacts and value.

## Figures and Tables

**Table 1:** Main stakeholders of the Treborth Botanic Garden (TBG).

Main Stakeholders	Included	Reason for Inclusion
Bangor University	Inputs included	Bangor University staff are responsible for running TBG and its volunteer programme.
TBG volunteers	Outcomes included	Well-being benefits to TBG volunteers are the primary focus of this study.
NHS Wales	Outcomes included	NHS Wales may benefit if the positive changes experienced by Treborth volunteers reduce mental health service use.

**Table 3:** Baseline characteristics of Treborth Botanic Garden (TBG) volunteers (complete cases only).

	Participants (TBG Volunteers) (*n* = 35)
Gender	Male 44%, 56% female
Age	Mean age of 55 years, ranging from 20 to 91 years
Ethnicity	88% White British, 6% unstated, 3% Black British, 3% Mixed
Mean time volunteering at TBG	11 years, 4 months (112 months)
Type of volunteer	74% existing volunteers, 20% student volunteers, 6% staff volunteers

**Table 4:** A comparison of outcome measures at the baseline and follow-up (complete cases only).

Volunteer Outcomes	Outcome Measure	# of Complete Cases	# of Volunteers Reporting Positive Change	Mean at Baseline ± SD	Mean at Follow-Up ± SD	Mean % Difference ± SD
Good overall health	ICECAP-A	33	23/33	0.805 ± 0.149	0.891 ± 0.077	+9 ± 14
Mental well-being	SWEMWBS	35	27/35	22 ± 6	25 ± 4	+9 ± 16
Basic psychological need satisfaction	BPNSFS	32	16/32	22 ± 5	24 ± 4	+5 ± 17
Basic psychological need frustration	BPNSFS	32	21/32	15 ± 5	12 ± 4	−11 ± 17
Connection to nature	NCI	34	10/34	75 ± 26	78 ± 27	+3 ± 12

**Table 5:** Related sample sign test results for volunteer outcomes.

Volunteer Outcomes	Outcome Measure	N	Standardised Test Statistic	Standard Error	*p*-Value
Good overall health	ICECAP-A	33	3.726	2.550	<0.001 ***
Mental well-being	SWEMWBS	35	3.482	2.872	<0.001 ***
Basic psychological need satisfaction	BPNSFS	32	0.981	2.550	0.327
Basic psychological need frustration	BPNSFS	32	−2.514	2.784	0.012 *
Connection to nature	NCI	34	0.671	2.236	0.503

* *p*-value < 0.05, 95% CI; *** *p*-value < 0.001, 95% CI.

**Table 6:** Social value per participant calculated using SVC methodology.

Outcomes	Outcome Measure	Quantity Improved	Financial Value	Total Social Value for Volunteers	Social Value per Volunteer (*n* = 35)
Volunteering	Regular volunteering question	28/35 reported volunteering regularly for two months or more	GBP 5344 per person per year	GBP 149,632	GBP 4275
Good Overall Health	ICECAP-A	21/33 reported an improvement > 10%	GBP 20,791 per person per year	GBP 436,611	GBP 12,475
Total Social Value				GBP 586,243	GBP 16,750

**Table 7:** SVC social value outcomes adjusted for deadweight, attribution, and displacement.

Outcomes	Total Social Value	Deadweight	Attribution	Displacement	Total Social Value	Total Social Value per Volunteer
Volunteering	GBP 149,632	10% (×0.90)	57% (×0.43)	43% (×0.57)	GBP 33,007	GBP 943 (*n* = 35)
Good overall health	GBP 436,611	10% (×0.90)	57% (×0.43)	43% (×0.57)	GBP 96,312	GBP 2752 (*n* = 35)
Social impact	GBP 586,243				GBP 129,319	GBP 3695

**Table 8:** TBG volunteers’ NHS mental health resource use.

	Average Number of Visits 3 Months Before Volunteering	Average Number of Visits in 3 Months prior to Follow-Up	Difference in Visits	Cost per Visit	Cost Saving per 3 Months	Cost Saving per 12 Months
Psychotherapist	12	0	12	GBP 216 per visit ^2^	GBP 2592	GBP 10,368
Counselling	12	12	0	GBP 75 per visit ^1^	GBP 0	GBP 0
Psychologist	7	17	−10	GBP 125 per visit ^1^	GBP −1250	GBP −5000
Mental health nurse	5	5	0	GBP 21 per visit ^1^	GBP 0	GBP0
Total cost saving					GBP 1342	GBP 5368
Total cost saving per volunteer (*n* = 27)						GBP 199

^1^ NHS Reference Costs 2021/22; ^2^ PSSRU 2022.

**Table 9:** Costs attributed to TBG volunteering.

Cost Category	Time	Annual Costs	Daily Cost	Cost per Volunteer (*n* = 96)
Annual staff costs				
Time recruiting, planning, and managing volunteers	160 h/mo	GBP 27,396	GBP 75	GBP 285
Total staffing costs		GBP 27,396		
Annual running costs				
Building lease and maintenance costs		GBP 10,000	GBP 27	GBP 104
Utilities (heating, water, etc.)		GBP 96,000	GBP 263	GBP 1000
Average total equipment cost (tools, etc.)		GBP 2000	GBP 5	GBP 21
Average total material cost (compost, plants, etc.)		GBP 3000	GBP 8	GBP 31
Catering for volunteers (refreshments)		GBP 600	GBP 2	GBP 6
Insurance		GBP 600	GBP 2	GBP 6
Total running costs		GBP 112,200	GBP 307	GBP 1168
Apportioned for volunteers (37%)		GBP 41,514	GBP 114	GBP 432
Annual total costs (staff costs + running costs)				
Total		GBP 68,910	GBP 189	GBP 717

**Table 10:** SROI ratios using SVC and MHVC.

	SVC	MHSVC
Total social value per person per year	GBP 3695	GBP 2684
NHS cost savings per person per year	GBP 199	GBP 199
Total monetary value per person per year	GBP 3894	GBP 2883
Total cost per person per year	GBP 717	GBP 717
SROI ratio for volunteers	GBP 5.43: GBP 1	GBP 4.02: GBP 1

## Data Availability

The datasets presented in this article are not readily available because of privacy reasons. Requests to access the datasets should be directed to the corresponding author.

## Appendix A

**Table A1:** Social value per TBG volunteer calculated using MHSVC methodology.

ID	SWEMWBS Score at Baseline	Social Value at Baseline	SWEMWBS Score at Follow-Up	Social Value at Follow-Up	SWEMWBS Difference (T2-T1)	Social Value Difference (T2-T1)	Adjusted for Deadweight
1	33	GBP 26,175	35	GBP 26,793	2	GBP 618	GBP 451
2	29	GBP 25,480	29	GBP 25,480	0	GBP 0	GBP 0
3	7	0	21	GBP 21,049	14	GBP 21,049	GBP 15,366
4	23	GBP 22,944	25	GBP 24,225	2	GBP 1281	GBP 935
5	23	GBP 22,944	26	GBP 24,225	3	GBP 1281	GBP 935
6	21	GBP 21,049	22	GBP 21,049	1	GBP 0	GBP 0
7	19	GBP 17,561	22	GBP 21,049	3	GBP 3488	GBP 2546
8	20	GBP 17,561	24	GBP 22,944	4	GBP 5383	GBP 3930
9	26	GBP 24,225	30	GBP 25,480	4	GBP 1255	GBP 916
10	25	GBP 24,225	25	GBP 24,225	0	GBP 0	GBP 0
11	24	GBP 22,944	26	GBP 24,225	2	GBP 1281	GBP 935
12	32	GBP 25,856	20	GBP 17,561	−12	GBP −8295	GBP −6055
13	25	GBP 24,225	26	GBP 24,225	1	GBP 0	GBP 0
14	24	GBP 22,944	23	GBP 22,944	−1	GBP 0	GBP 0
15	21	GBP 21,049	24	GBP 22,944	3	GBP 1895	GBP 1383
16	35	GBP 26,793	34	GBP 26,175	−1	GBP −618	GBP −451
17	21	GBP 21,049	24	GBP 22,944	3	GBP 1895	GBP 1383
18	25	GBP 24,225	27	GBP 24,877	2	GBP 652	GBP 476
19	19	GBP 17,561	26	GBP 24,225	7	GBP 6664	GBP 4865
20	15	GBP 9639	25	GBP 24,225	10	GBP 14,586	GBP 10,648
21	24	GBP 22,944	26	GBP 24,225	2	GBP 1281	GBP 935
22	23	GBP 22,944	29	GBP 25,480	6	GBP 2536	GBP 1851
23	7	0	22	GBP 21,049	15	GBP 21,049	GBP 15,366
24	17	GBP 12,255	26	GBP 24,225	9	GBP 11,970	GBP 8738
25	19	GBP 17,561	22	GBP 21,049	3	GBP 3488	GBP 2546
26	26	GBP 24,225	25	GBP 21,049	−1	GBP 0	GBP 0
27	11	0	29	GBP 25,480	18	GBP 25,480	GBP 18,600
28	21	GBP 21,049	25	GBP 24,225	4	GBP 3176	GBP 2318
29	19	GBP 17,561	20	GBP 17,561	1	GBP 0	GBP 0
30	21	GBP 21,049	25	GBP 24,225	4	GBP 3176	GBP 2318
31	21	GBP 21,049	24	GBP 22,944	3	GBP 1895	GBP 1383
32	26	GBP 24,225	20	GBP 17,561	−6	GBP −6664	GBP −4865
33	26	GBP 24,225	25	GBP 24,225	−1	GBP 0	GBP 0
34	20	GBP 17,561	21	GBP 21,049	1	GBP 3488	GBP 2546
35	19	GBP 17,561	23	GBP 22,944	4	GBP 5383	GBP 3930
Total		GBP 682,658		GBP 808,155		GBP 128,673	GBP 93,930
Total social value per volunteer (*n* = 35)							GBP 2684

## References

[1] Carr M.J., Steeg S., Webb R.T. (2021). Effects of the COVID-19 pandemic on primary care-recorded mental illness and self-harm episodes in the UK: A population-based cohort study. Lancet Public Health.

[2] Mahase E. (2022). NHS England sets out ambitious new mental health access standards to deal with pandemic demand. BMJ.

[3] Jackson S.E., Brown J., Shahab L., McNeill A., Munafò M.R., Brose L. (2023). Trends in psychological distress among adults in England, 2020–2022. JAMA Netw. Open.

[4] Skivington K., Matthews L., Simpson S.A., Craig P., Baird J., Blazeby J.M., Boyd K.A., Craig N., French D.P., McIntosh E. (2021). A new framework for developing and evaluating complex interventions: Update of Medical Research Council guidance. BMJ.

[5] Bragg R., Leck C. (2017). Good Practice in Social Prescribing for Mental Health: The Role of Nature-Based Interventions.

[6] Shanahan D.F., Astell–Burt T., Barber E.A., Brymer E., Cox D.T., Dean J., Depledge M., Fuller R.A., Hartig T., Irvine K.N. (2019). Nature–based interventions for improving health and well-being: The purpose, the people and the outcomes. Sports.

[7] Wilkie S., Davinson N. (2021). Prevalence and effectiveness of nature-based interventions to impact adult health-related behaviours and outcomes: A scoping review. Landsc. Urban Plan..

[8] Pretty J., Barton J. (2020). Nature-Based Interventions and Mind-Body Interventions: Saving Public Health Costs Whilst Increasing Life Satisfaction and Happiness. Int. J. Environ. Res. Public Health.

[9] Gritzka S., MacIntyre T.E., Dörfel D., Baker-Blanc J.L., Calogiuri G. (2020). The effects of workplace nature-based interventions on the mental health and well-being of employees: A systematic review. Front. Psychiatry.

[10] Wood C.J., Polley M., Barton J.L., Wicks C.L. (2022). Therapeutic community gardening as a green social prescription for mental ill-health: Impact, barriers, and facilitators from the perspective of multiple stakeholders. Int. J. Environ. Res. Public Health.

[11] Coventry P.A., Brown J.E., Pervin J., Brabyn S., Pateman R., Breedvelt J., Gilbody S., Stancliffe R., McEachan R., White P.L. (2021). Nature-based outdoor activities for mental and physical health: Systematic review and meta-analysis. SSM Popul. Health.

[12] Thomas T., Aggar C., Baker J., Massey D., Thomas M., D’Appio D., Brymer E. (2022). Social prescribing of nature therapy for adults with mental illness living in the community: A scoping review of peer-reviewed international evidence. Front. Psychol..

[13] Odeh R., Diehl E.R., Nixon S.J., Tisher C.C., Klempner D., Sonke J.K., Colquhoun T.A., Li Q., Espinosa M., Perdoma D. (2022). A pilot randomized controlled trial of group-based indoor gardening and art activities demonstrates therapeutic benefits to healthy women. PLoS ONE.

[14] Chalmin-Pui L.S., Griffiths A., Roe J., Heaton T., Cameron R. (2021). Why garden?–Attitudes and the perceived health benefits of home gardening. Cities.

[15] Kingsley J.Y., Townsend M., Henderson-Wilson C. (2009). Cultivating health and well-being: Members’ perceptions of the health benefits of a Port Melbourne community garden. Leis. Stud..

[16] Cochran S., Minaker L. (2020). The value in community gardens: A return on investment analysis. Can. Food Stud..

[17] Public Health England (2020). Improving Access to Greenspace: A New Review for 2020.

[18] Edwards R.T., Lawrence C.L. (2024). Health Economics of Well-Being and Well-Becoming Across the Life-Course.

[19] Jiang D., Warner L.M., Chong A.M.L., Li T., Wolff J.K., Chou K.L. (2021). Benefits of volunteering on psychological well-being in older adulthood: Evidence from a randomized controlled trial. Aging Ment. Health.

[20] Pettigrew S., Jongenelis M.I., Jackson B., Warburton J., Newton R.U. (2020). A randomized controlled trial and pragmatic analysis of the effects of volunteering on the health and well-being of older people. Aging Clin. Exp. Res..

[21] Yang J., Matz C. (2022). A latent deprivation perspective: Mechanisms linking volunteering to mental health in later life. Int. J. Aging Hum. Dev..

[22] Huo M., Miller L.M.S., Kim K., Liu S. (2021). Volunteering, self-perceptions of aging, and mental health in later life. Gerontologist.

[23] de Wit A., Qu H., Bekkers R. (2022). The health advantage of volunteering is larger for older and less healthy volunteers in Europe: A mega-analysis. Eur. J. Ageing.

[24] McGarvey A., Jochum V., Davies J., Dobbs J., Honrung L. (2019). Time Well Spent: A National Survey on the Volunteer Experience.

[25] Kim E.S., Whillans A.V., Lee M.T., Chen Y., VanderWeele T.J. (2020). Volunteering and subsequent health and well-being in older adults: An outcome-wide longitudinal approach. Am. J. Prev. Med..

[26] Granerud A., Eriksson B.G. (2014). Mental health problems, recovery, and the impact of green care services: A qualitative, participant-focused approach. Occup. Ther. Ment. Health.

[27] Cutcliffe J.R., Travale R. (2016). Unearthing the theoretical underpinnings of “Green Care” in mental health and substance misuse care: Theoretical underpinnings and contemporary clinical examples. Issues Ment. Health Nurs..

[28] Güntert S.T., Strubel I.T., Kals E., Wehner T. (2016). The quality of volunteers’ motives: Integrating the functional approach and self-determination theory. J. Soc. Psychol..

[29] Wu Y., Li C. (2019). Helping Others Helps? A Self-Determination Theory Approach on Work Climate and Well-being among Volunteers. Appl. Res. Qual. Life.

[30] Pritchard A., Richardson M., Sheffield D., McEwan K. (2020). The relationship between nature connectedness and eudaimonic well-being: A meta-analysis. J. Happiness Stud..

[31] Kuo M. (2015). How might contact with nature promote human health? Promising mechanisms and a possible central pathway. Front. Psychol..

[32] Bratman G.N., Hamilton J.P., Hahn K.S., Daily G.C., Gross J.J. (2015). Nature experience reduces rumination and subgenual prefrontal cortex activation. Proc. Natl. Acad. Sci. USA.

[33] McDonnell A.S., Strayer D.L. (2024). The influence of a walk in nature on human resting brain activity: A randomized controlled trial. Sci. Rep..

[34] Deidda M., Geue C., Kreif N., Dundas R., McIntosh E. (2019). A framework for conducting economic evaluations alongside natural experiments. Soc. Sci. Med..

[35] Nicholls J., Lawlor E., Neitzert E., Goodspeed T. (2012). A Guide to Social Return on Investment.

[36] Edwards R.T., McIntosh E. (2019). Applied Health Economics for Public Health Practice and Research.

[37] Trotter L., Rallings Adams M.-K. (2017). Valuing Improvements in Mental Health: Applying the Well-Being Valuation Method to WEMWBS.

[38] Flynn T.N., Huynh E., Peters T.J., Al-Janabi H., Clemens S., Moody A., Coast J. (2015). Scoring the Icecap-a Capability Instrument. Estimation of a UK General Population Tariff. Health Econ..

[39] Al-Janabi H., Flynn T.N., Peters T.J., Bryan S., Coast J. (2015). Test–Retest Reliability of Capability Measurement in the UK General Population. Health Econ..

[40] Stewart-Brown S.L., Platt S., Tennant A., Maheswaran H., Parkinson J., Weich S., Tennant R., Taggart F., Clarke A. (2011). The Warwick-Edinburgh Mental Well-being Scale (WEMWBS): A valid and reliable tool for measuring mental well-being in diverse populations and projects. J. Epidemiol. Community Health.

[41] Mack D.E., Vo K.T., Wilson P.M. (2024). The Long and Short-Form Warwick-Edinburgh Mental Well-Being Scale: A Reliability Generalization Meta-Analysis. J. Happiness Stud..

[42] Tennant R., Hiller L., Fishwick R., Platt S., Joseph S., Weich S., Parkinson J., Secker J., Stewart-Brown S. (2007). The Warwick-Edinburgh Mental Well-being Scale (WEMWBS): Development and validation. Health Qual. Life Outcomes.

[43] Trotter L., Vine J., Leach M., Fujiwara D. (2014). Measuring the Social Impact of Community Investment: A Guide to Using the Well-Being Valuation Approach.

[44] Hartfiel N., Gittins H., Morrison V., Wynne-Jones S., Dandy N., Edwards R.T. (2023). Social Return on Investment of Nature-Based Activities for Adults with Mental Well-being Challenges. Int. J. Environ. Res. Public Health.

[45] Jones K., Weatherly H., Birch S., Castelli A., Chalkley M., Dargan A., Forder J., Gao M., Hinde S., Markham S. (2023). Unit Costs of Health and Social Care 2022 Manual.

[46] NHS England (2023). Patient Level Activity and Costing, 2021–2022. https://digital.nhs.uk/data-and-information/publications/statistical/patient-level-activity-and-costing/2021-22/relationship-to-national-cost-collection.

[47] Van der Kaap-Deeder J., Soenens B., Ryan R.M., Vansteenkiste M. (2020). Manual of the Basic Psychological Need Satisfaction and Frustration Scale (BPNSFS).

[48] Heissel A., Pietrek A., Flunger B., Fydrich T., Rapp M.A., Heinzel S., Vansteenkiste M. (2018). The Validation of the German Basic Psychological Need Satisfaction and Frustration Scale in the Context of Mental Health. Eur. J. Health Psychol..

[49] Vansteenkiste M., Ryan R.M. (2013). On psychological growth and vulnerability: Basic psychological need satisfaction and need frustration as a unifying principle. J. Psychother. Integr..

[50] Richardson M., Hunt A., Hinds J., Bragg R., Fido D., Petronzi D., Barbett L., Clitherow T., White M. (2019). A Measure of Nature Connectedness for Children and Adults: Validation, Performance, and Insights. Sustainability.

[51] Milyavskaya M., Koestner R. (2011). Psychological needs, motivation, and well-being: A test of self-determination theory across multiple domains. Personal. Individ. Differ..

[52] Ryan R.M., Deci E.L. (2020). Self-determination theory and the facilitation of intrinsic motivation, social development, and well-being. Am. Psychol..

[53] Schoen V., Caputo S., Blythe C. (2020). Valuing physical and social output: A rapid assessment of a London community garden. Sustainability.

[54] Bojke L., Schmitt L., Lomas J., Richardson G., Weatherly H. (2018). Economic Evaluation of Environmental Interventions: Reflections on Methodological Challenges and Developments. Int. J. Environ. Res. Public Health.

[55] Parkinson J.A., Eccles K.E. (2014). Positive impact by design: The Wales Centre for Behaviour Change. J. Posit. Psychol..

